# Systematic Chemical Analysis Approach Reveals Superior Antioxidant Capacity via the Synergistic Effect of Flavonoid Compounds in Red Vegetative Tissues

**DOI:** 10.3389/fchem.2018.00009

**Published:** 2018-02-02

**Authors:** Xiaoxiao Qin, Yanfen Lu, Zhen Peng, Shuangxi Fan, Yuncong Yao

**Affiliations:** Technology Industry Group, Beijing Key Laboratory of New Technology in Agricultural Application, National Demonstration Center for Experimental Plant Production Education, Beijing Collaborative Innovation Center for Eco-Environmental Improvement with Forestry Fruit Trees, Plant Science and Technology College, Beijing University of Agriculture, Beijing, China

**Keywords:** systematic approach, synergistic antioxidant, flavonoids, red leaves, *Malus* crabapple

## Abstract

The flavonoid system comprises an abundance of compounds with multiple functions; however, their potential synergism in antioxidant function remains unclear. We established an approach using ever-red (RL) and ever-green leaves (GL) of crabapple cultivars during their development to determine interrelationships among flavonoid compounds. RL scored significantly better than GL in terms of the type, composition, and diversity of flavonoids than GL. Principal component analysis predicted flavonoids in RL to have positive interaction effects, and the total antioxidant capacity was significantly higher than the sum of antioxidant capacities of the individual compounds. This synergy was verified by the high antioxidant capacity in rat serum after feeding on red leaves. Our findings suggest that the synergistic effect is a result of the high transcription levels regulated by McMYBs in RL. In summary, individual flavonoids cooperate in a flavonoid system, thus producing a synergistic antioxidant effect, and the approach used herein can provide insights into the roles of flavonoids and other compounds in future studies.

## Introduction

Flavonoids are the most abundant class of polyphenol compounds in the human diet and are widely found in grains, vegetables, fruits, and their processed products (wine, juice) and health foods (Bondonno et al., 2012; Czank et al., 2013). The basic flavonoid structure consists of A and B benzene rings, an oxygen-containing pyran C ring and a polyhydroxyl structure, which enables a single compound to have strong and diverse biological functions, including antioxidant, anti-aging, and anticarcinogenic capacities (Tsuji et al., 2013). For example, catechin from green tea (Chyu et al., 2004), phloridzin from *Malus* plants (Nair et al., 2014), and anthocyanins from berries (Bagchi et al., 2004) have been shown to reduce atherosclerotic lesions, to have anticancer effects, and to have anti-oxidant functions, respectively. Furthermore, various flavonoid monomers have been actively developed as drugs used in human disease control. For example, the quercetin monomer can regulate hyperthyroidism (Panda and Kar, 2007).

Thousands of flavonoid phytochemicals have been identified throughout the plant kingdom (Crozier et al., 2009). The main sub-classes of flavonoids are flavones, flavanones, isoflavones, flavonols and their glycosides, flavanonols, flavan-3-ols, and anthocyanidins, while the minor components include flavan-3,4-diols, biflavones, dihydrochalcones, and flavans (Pietta, 2000; Cushnie and Lamb, 2005; Crozier et al., 2009). Flavonoids cooperate to form a biochemical reaction system. When considering individual flavonoid compounds as drugs for disease treatment and stress release, it is important to note that although these flavonoid compounds are present in most dietary sources and are beneficial for human health, how exactly the flavonoid system functions and the effects of intake of these compounds in animals remain poorly understood.

The flavonoid system has several properties. First, there is a top-down metabolic flux from flavones, via flavonols, to anthocyanidins in the phenylpropanoid biosynthesis pathway (Shirley, 1996). Second, this system contains several important metabolic branches, from dihydrochalcones to phloridzin and its glycoside, dihydroflavonols to flavonols (e.g., quercetin, kaempferol, rutin, and their ligands), leucoanthocyanidins to flavan-3-ols (e.g., catechinic acid, epicatechin), and anthocyanidins to anthocyanins (Falcone Ferreyra et al., 2012; Henry-Kirk et al., 2012). Third, there are complex interrelations among compounds, including positive and negative feedback regulation in metabolic processes (Yin et al., 2012). Fourth, this system changes constantly in response to environmental and developmental cues. Fifth, most compounds have powerful biological functions, such as antioxidant, anti-aging, and anti-cancer capacities (Czank et al., 2013; Sáez-Ayala et al., 2015; Srinivasan et al., 2016). Moreover, different plants, organs, and tissues have different compositions and contents. For example, flavonoid compounds are significantly more abundant in red (fruit peels, leaves, berries) than in green tissues (Revilla and Ryan, 2000; Tsao et al., 2003; Tian et al., 2015). These contents are associated with the transcriptional regulation of structural genes in the flavonoid biosynthesis pathway. Flavonoids are synthesized from three molecules of malonyl CoA and one molecule of p-coumaroyl CoA via the phenylpropanoid pathway. Anthocyanin accumulation in ever-red leaf crabapple (*Malus* spp. “Royalty”) is regulated by the transcription factor *Mc*MYB10, which activates *McF3*′*H* and other structural genes involved in anthocyanin biosynthesis (Tian et al., 2015). In white Chilean strawberry (*Fragaria chiloensis* ssp. *chiloensis* f. chiloensis) fruit, down regulation of *FcMYB1* expression results in the up-regulation of *ANS* and strong repression of anthocyanidin reductase (*ANR*) and leucoanthocyanidin reductase (*LAR*) transcript accumulation (Salvatierra et al., 2013). In developing seedlings of *Arabidopsis thaliana, AtMYB11, AtMYB12*, and *AtMYB111* control flavonol accumulation through transcriptional regulation of their target genes (Stracke et al., 2007; Pandey et al., 2014).

Free radicals, as strong oxidizers, can cause degenerative diseases and stress, such as cancer, aging, and apoptosis. The antioxidant' properties of flavonoids have been well-described (Candeias et al., 1993; Roleira et al., 2015). The mechanisms underlying antioxidant activities of flavonoids include (1) suppression and scavenging of reactive oxygen species by inhibiting enzymes or chelating trace elements involved in free-radical production; (2) inhibition of lipid peroxidation by decreasing malondialdehyde (MDA) levels; and (3) up-regulation or protection of antioxidant defenses by stimulating the activities of antioxidant enzymes, such as superoxide dismutase (SOD), catalase (CAT), peroxidase (POD), ascorbate peroxidase (APX), and glutathione peroxidase (GPX) (Symons and Gutteridge, 1998). Procyanidins have been found to decrease MDA levels, maintain a high reduced/oxidized glutathione ratio, and increase CAT/SOD and GPX/SOD ratios as well as glutathione reductase and glutathione transferase activities (Roig et al., 2002). The actual antioxidant capacity, depends on the concentration of flavonoid compounds and the positive interaction/synergistic effects among the antioxidants in a mixture (Hernandez et al., 2009). However, synergistic interactions of flavonoid system compounds with respect to antioxidant capacity remain unclear.

To explore such synergistic effects, we focused on the *Malus* crabapple, which belongs to the family Rosaceae, *Malus Mill*. *Malus* crabapple originated in Europe and have been cultivated worldwide for 240 years, and the genome of crabapple is highly similar to that of *Malus domestica* (Zhang, 2007; Velasco et al., 2010). In this study, for comparison, we selected a *Malus* cultivar, *M*. spp. “Royalty,” with typical ever-red leaves, and *M*. spp. “Flame,” with ever-green traits and leaves similar to those of *M. domestica*. In our previous AFLP-based genetic study, we found that both cultivars are closely related (Zhang, 2007). The highly abundant flavonoids in crabapple leaves have antitumor activity and have been suggested to represent an excellent source of antioxidants for use as food additives (Qin et al., 2015). Our preliminary study revealed that the diversity and contents of phenolic compounds in “Royalty” leaves are greater than those in *M. domestica* leaves, and *Malus* crabapple represents a useful model system as one of the most economically important ornamental apple germplasm resources owing to the high flavonoid levels in leaves, flowers, and fruits (Tian et al., 2011). Here, we developed a systematic approach to evaluate the potential synergistic antioxidant effect of flavonoids in crabapple, including: (1) identification of individual compound classes; (2) quantification of the temporal dynamics of all compounds at various leaf developmental stages; (3) estimation of diversity properties of the flavonoid system; (4) clustering of leaves based on diversity indices and selection of optimal leaves for following measurements and/or applications; (5) analysis of the effects, contributions, and interrelations of individual compounds to identify predominant compounds using principal component analysis (PCA); (6) exploration of the molecular mechanism of flavonoid system establishment; (7) detection of the antioxidant capacity of flavonoid compounds in on-line and off-line systems to compare differences in antioxidant between total antioxidant capacity (T-AOC) and the sum of capacities of individual compounds; (8) verification of the antioxidant capacity in animal experiments.

## Results

### Types and compositions of flavonoid compounds

To identify the flavonoid compounds in crabapple red leaves (RL) and green leaves (GL), we subjected leaf extracts to high-performance liquid chromatography coupled with tandem mass spectrometry (HPLC-MS) and compared the findings to the standards and reported references. In total, 19 compounds were identified (Text S1), including 16 flavonoid compounds, one phenolic acid and two terpenoids (Table 1 and Supplementary Figure S1). RL contained 17 of the 19 compounds, and GL contained 16 of the 19 compounds, with 14 compounds in common. P2, P4, and P7 were detected only in RL, while P6 and P19 were detected only in GL.

**Table 1:** Main classes of compounds identified by LC-UV-ESI-MS^2^ in crabapple leaves.

**No**.	**MW**	**[M-H]^−a^**	**MS^2^[M-H]-(m/z)^a^**	**Putative identify**	**RL**	**GL**	**Classification**
1	626	625	[625]:463,301	Quercetin-3-O-diglucoside (P1)^c^(Sanchez-Rabaneda et al., 2004)	√	√	Flavonol
2	449	–	[449]:287	Cyanidin-3-O-galactoside (P2)^b^	√	ND	Anthocyanin
3	354	353	[353]:191,173,179	4-O-caffeoylquinic acid (P3)^b^	√	√	Phenolic acid
4	466	465	[465]:285,241	Taxifolin-3-O-glucoside (P4)^b^	√	ND	Dihydroflavonol
5	578	577	[577]:451,425,407	Procyanidin B2 (P5)^b^	√	√	Flavan-3-ols
6	518	517	[517]:385	Xyloside roseoside (P6)	ND	√	Terpenoids
7	450	449	[449]:269	Astilbin (P7)^b^	√	ND	Dihydroflavonol
8	386	385	[385]:223	Roseoside (P8)^c^(Winterhalter et al., 1994)	√	√	Terpenoids
9	290	289	[289]:245,205	(-)–Epicatechin (P9)^b^	√	√	Flavan-3-ols
10	610	609	[609]:565,301	Rutin (P10)^b^	√	√	Flavonol
11	464	463	[463]:301	Quercetin-3-O-glucoside (P11)^b^	√	√	Flavonol
12	464	463	[463]:301	Quercetin-7-O-glucoside (P12)	√	√	Flavonol
13	434	433	[433]:301	Quercetin-3-O-arabinoside (P13)^b^	√	√	Flavonol
14	506	505	[505]:463,301	Acetyl quercetin-3-O-glucoside(P14)	√	√	Flavonol
15	506	505	[505]:463,301	Acetyl quercetin-7-O-glucoside (P15)	√	√	Flavonol
16	434	433	[433]:301	Quercetin-7-O-arabinoside (P16)	√	√	Flavonol
17	448	447	[447]:301	Quercetin-3-O-rhamnoside (P17)^c^(Lommen et al., 2000)	√	√	Flavonol
18	436	435	[436]:273	Phloridzin (P18)^b^	√	√	Dihydrochalcone
19	594	593	[593]:447:285	Luteolin-5-O-rutinoside (P19)	ND	√	Flavone

*MW, molecular weight*.

a *Obtained by ion trap mass spectrometry*.

b *Identification confirmed by direct comparison with standards*.

c *Identification confirmed by reference compounds in the plant sample*.

*P1–P19, serial number of the identified compounds, used henceforth*.

*RL and GL, the ever-red and ever-green leaves, respectively from M.cv. “Royalty” and M.cv. “Flame” crabapple, used henceforth*.

*ND, none detected; √, detected*.

**Figure 1:**
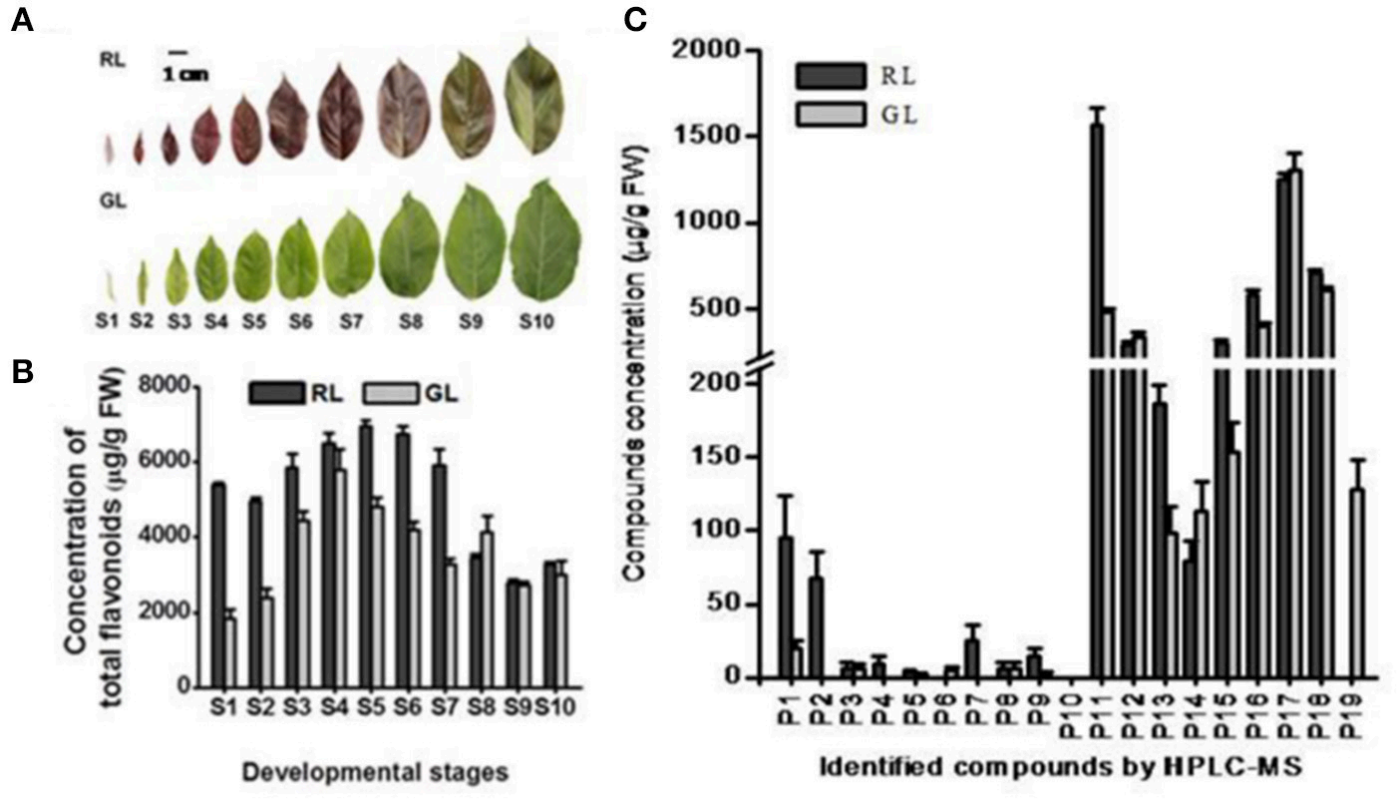
Analysis of phenotypes and compound compositions detected by HPLC in *Malus* ever-red (RL) and ever-green leaves (GL). **(A)** Phenotypes of leaves at 10 developmental stages of RL and GL. Scale bar represents 1.0 cm. **(B)** HPLC analysis of total detected compounds at the 10 developmental stages in RL and GL. The data (mean ± SD) are the sum for each detected compound. S1–S10, the leaves at the 1st to 10th nodes on the annual shoot from the tip toward the base, represent the 10 leaf developmental stages. **(C)** HPLC analysis of each compound detected in the leaves at the 10 developmental stages of RL and GL. The data show the average values (mean ± SD) of the compounds in S1–S10. P1–P19 is the same as in Table 1.

The concentrations of total flavonoid compounds were significantly higher in RL than in GL at the S1–S7 and S10 leaf nodes (representing leaf developmental stages, Figure 1B). The average contents of flavonoid compounds P1, P2, P4, P7, P9, P11, P13, P15, P16, and P18 were significantly higher in RL than in GL (Figure 1C) during all leaf developmental stages (Figure 1C). The dynamics of the changes in the individual flavonoid compounds differed significantly between RL and GL (Supplementary Figure S2). The profiles of these compounds may reflect differences in the distribution and composition of flavonoid metabolites in the leaves of the two cultivars. Luteolin-5-O-rutinoside, a flavone produced from the direct precursor naringenin, by flavone synthase (FNS), was detected only in GL.

### Properties of flavonoid systems

To determine the diversity of the flavonoid compounds in the leaves, we analyzed the diversity (H), dominance (D), and evenness (E) indices. H differed significantly between RL and GL, with high diversity during early leaf developmental stages. D was significantly higher for RL than for GL, except for S4. E also differed significantly between RL and GL in the S1, S2, S5, S6, S8, S9 developmental stages (Table 2). The three indices for S1, S2, S6, and S8 leaves showed significant differences between RL and GL. These results indicated that the diversity of the complex flavonoid systems in RL and GL throughout leaf development differs.

**Table 2:** The diversity properties of flavonoid compounds in crabapple leaves.

**Stages**	**Diversity (H)**		**Dominance (D)**		**Evenness (E)**	
	**RL**	**GL**	**RL**	**GL**	**RL**	**GL**
S1	2.9456^a^^**^	2.7475^c^	0.8239^b^^**^	0.7879^d^	0.7207^a^^**^	0.6869^c^
S2	2.9568^a^^**^	2.7280^c^	0.8283^a^^**^	0.8066^c^	0.7234^a^^**^	0.682^cd^
S3	2.9622^a^	2.9451^a^	0.8313^a^^**^	0.8446^a^	0.7247^a^	0.7363^a^
S4	2.8721^b^	2.8487^b^	0.8179^c^	0.8188^b^	0.7027^b^	0.7122^b^
S5	2.8428^c^^**^	2.6414^d^	0.8141^d^^**^	0.7837^d^	0.6955^bc^^**^	0.6761^d^
S6	2.8249^c^^**^	2.5476^e^	0.8085^e^^**^	0.7580^f^	0.6911^c^^**^	0.6520^e^
S7	2.6684^d^^**^	2.5509^e^	0.7845^f^^**^	0.7650^e^	0.6777^d^^*^	0.6529^e^
S8	2.8282^c^^**^	2.4763^f^	0.807^e^^**^	0.7425^g^	0.7239^a^^**^	0.6338^f^
S9	2.8379^c^^**^	2.5252^e^	0.8059^e^^**^	0.7876^d^	0.7268^a^^**^	0.6463^e^
S10	2.5220^e^	2.4764^f^	0.7632^g^^**^	0.7467^g^	0.6455^e^^*^	0.6339^f^

*S1–S10 is the same as in Figure 1*.

*Lower-case letters indicate significance differences among S1–S10 at P < 0.05 by Duncan's new multiple range test*.

** and * *indicate significance differences between RL and GL at P < 0.01 and P < 0.05 by t-test, respectively*.

**Figure 2:**
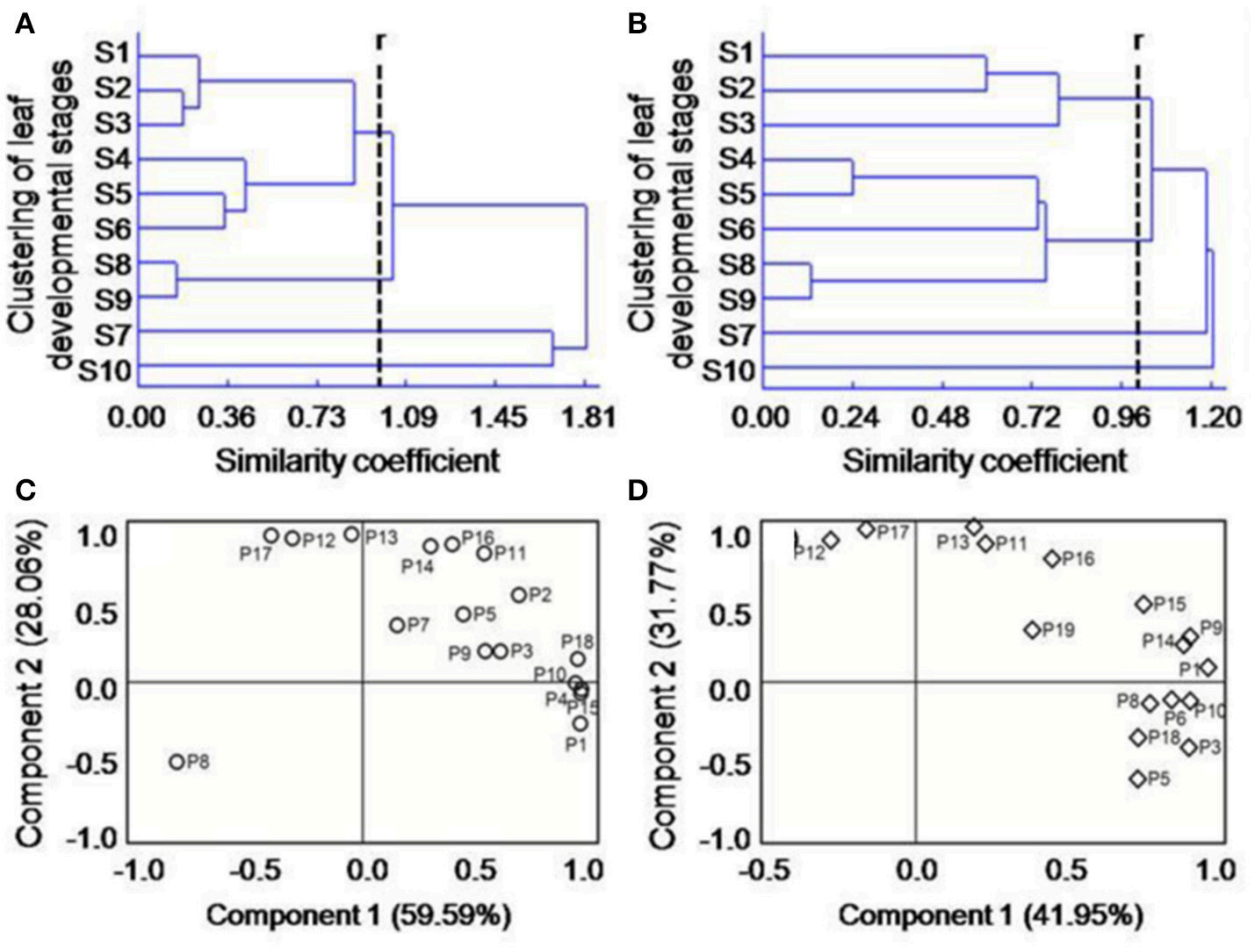
Clustering analysis of the diversity characteristics of flavonoid compounds in RL **(A)** and GL **(B)** and principal component analysis of various types of compounds in the flavonoid systems of RL **(C)** and GL **(D)**; *r* represents the similarity coefficient. P1-P19 in **(C,D)** are the same as in Table 1.

Based on the above indices, we conducted CA to identify groups with similar temporal diversity of the flavonoid composition. When the similarity coefficient r ≈ 1 (Chauhan and Vaish, 2012), these temporal patterns for flavonoid composition in RL and GL could be divided into four groups (Figures 2A,B). In RL, the groups were S1–S6, S7, S10, and S8–S9; in GL, the groups were S1–S3, S4–S9, S7, and S10. These results suggest that the leaves at different nodes in each group have obvious differences in the spatiotemporal pattern of flavonoid diversity. S1–S6 in RL and S4–S9 in GL were the groups encompassing the most nodes, suggesting the changes in flavonoids diversity followed a similar spatiotemporal pattern among these leaf groups. The S4, S5, and S6 leaves were comparable in both RL and GL, which may inform leaf material selection in future studies.

To determine the contribution rates (CRs) [usually used to analyze the effects of various factors on system functions; (Baerenfaller et al., 2012)] of the individual flavonoids to the function of the flavonoid system and the differences between the two cultivars, we performed PCA based on the combined data sets for each flavonoid compound plus the 10 developmental periods. For RL (Figure 2C), the first and second principal components (PCs) accounted for 87.65% of the total variance, where PC_1_ = 59.59% and PC_2_ = 28.06%. In PC_1_, the CRs of P1, P4, P10, P15, and P18 were the greatest, and in PC_2_, the CRs of P11, P12, P14, P16, and P17 were the greatest. For GL (Figure 2D), the first and second PCs accounted for 73.72% of the total variance, where PC_1_ = 41.95% and PC_2_ = 31.77%. In PC_1_, the CRs of P1, P3, P6, P9, P10, and P14 were the greatest, and in PC_2_, the CRs of P13, P16, and P17 were the greatest. These results indicate that the numbers of flavonoid compounds in PC_1_ and PC_2_ differ between RL and GL and that different compounds have different contribution to the respective flavonoid systems.

### Flavonoid biosynthesis related gene profiling

**Figure 3:**
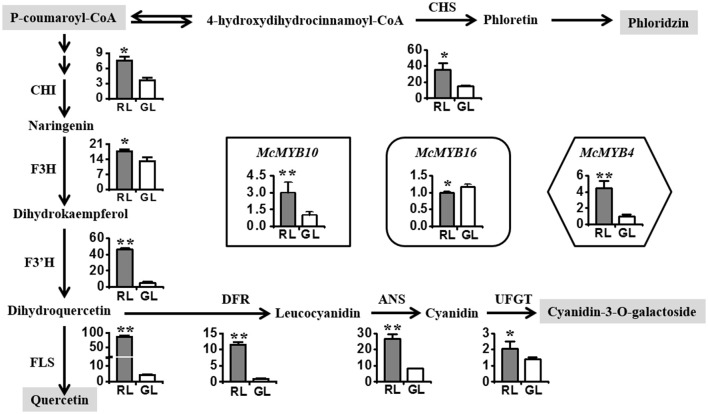
Analysis of the relative expression levels of the main structural genes and the transcription factor MYBs related to the flavonoid biosynthesis pathway in RL and GL. *CHS*, chalcone synthase; *CHI*, chalcone isomerase; *F3H*, flavanone 3β-hydroxylase; *F3*′*H*, flavonoid 3′-monooxygenase; *DFR*, dihydroflavonol 4-reductase; *ANS*, anthocyanidin synthase; *UFGT*, flavonoid 3-O-glycosyltransferase; *FLS*, flavonol synthase. *McMYB4, McMYB10*, and *McMYB16* are R2R3 transcription factors in crabapple. The data shown are the mean ± SD. ^**^ and ^*^ indicate significance at *P* < 0.01 and *P* < 0.05 by *t*-test, respectively.

To explore whether the differences in the levels of flavonoid compounds can be explained by different biosynthesis mechanisms in RL and GL, we selected the leaves at S6 (based on the above diversity index and CA results) to compare the expression levels of the main flavonoids biosynthetic genes using real-time PCR. The expression of *McCHS* was two-fold higher in RL than in GL (Figure 3), resulting in the accumulation of dihydrochalcone compounds, such as phloridzin dihydrates (Supplementary Figure S2). The expression levels of *McCHI, McF3H, McF3*′*H*, and *McFLS* were higher in RL than in GL (Figure 3). *McDFR, McANS*, and *McUFGT* showed higher expression in RL, leading to strong accumulation of cyanidin-3-O-galactoside (Supplementary Figure S2). *McMYB10*, encoding an R2R3 transcription factor (TF) involved in activating anthocyanin biosynthesis (Tian et al., 2015), showed significantly higher expression in RL than in GL. However, *McMYB16*, which is homologous to *FcMYB1*, a repressor of anthocyanin biosynthesis in strawberry fruit (Salvatierra et al., 2013), showed lower expression in RL than in GL. *McMYB4*, homologous to *AtMYB12*, which is involved in flavonol biosynthesis in Arabidopsis (Stracke et al., 2007), displayed a dramatically higher expression in RL than in GL.

### *In vitro* antioxidant capacity

**Figure 4:**
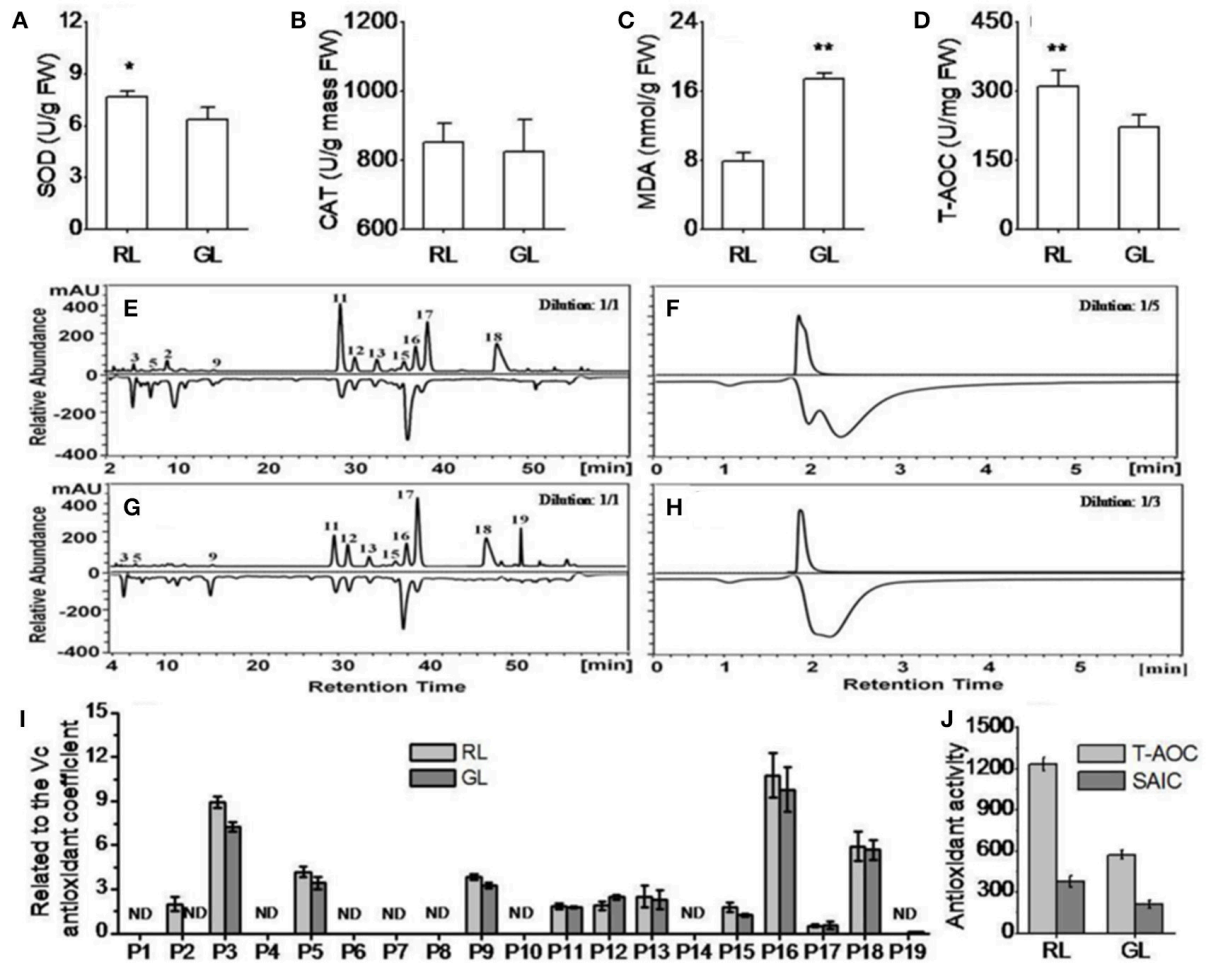
Antioxidant capacity of the detected flavonoid compounds in the extracts of RL and GL determined by HPLC-ABTS *in vitro*. **(A,B)** Superoxide dismutase (SOD) and catalase (CAT) activities. **(C,D)** Malondialdehyde (MDA) and total antioxidant capacity (T-AOC) levels. **(E,F)** Antioxidant activity (negative peaks) of each detected flavonoid compound (positive peaks) in RL and GL, respectively. **(G,H)** Total antioxidant activities (negative peaks) of the detected flavonoid compounds (positive peaks) in RL and GL, respectively. For each sample, the extract solution was diluted five-fold in RL and three-fold in GL. **(I)** Individual flavonoid compounds related to the Vc antioxidant coefficient in RL and GL. **(J)** Comparison of T-AOC (HPLC-ABTS) levels and sum of the antioxidant activities of the individual compounds (SAIC) (negative peak area) in RL and GL. The UV profile was monitored at 350 nm on the chromatogram, while the scavenging activity profile was determined using HPLC-ABTS and monitored at 517 nm (negative peaks). The values shown are the mean ± SD (*N* = 10/group). ^**^ and ^*^ indicate significance at *P* < 0.01 and *P* < 0.05 by *t*-test, respectively.

To compare the antioxidant capacity of RL and GL, we measured SOD and CAT activities, MDA content and T-AOC. SOD activity was significantly higher and MDA content significantly lower in RL than in GL, while CAT activity showed no significant difference between RL and GL. T-AOC was significantly higher in RL than in GL (Figures 4A–D).

To further explore the differences in antioxidant capacity for each flavonoid compound, we improved the HPLC with radical scavenging methods (HPLC-ABTS) system for crabapple leaf extracts based on screening results from the on-line HPLC-ABTS system (Figures 4E–H). The antioxidant activities of P3 (phenolic acid), P5, P9, P11, P13, P16, and P18 were significantly higher in RL than GL. P2 and P19 were detected only in RL and GL, respectively, and both had obvious antioxidant capacities (Supplementary Table S1). In addition, the antioxidant activities relative to the ascorbic acid (Vc) of P3, P5, P9, P11, P13, P15, P16, and P18 were higher in RL than in GL, while P12 activity was lower in RL than in GL. The differences in the activities of P3, P5, P9, P12, and P15 were statistically significant (Figure 4I). T-AOC (HPC-ABTS) was significantly higher than the sum of the antioxidant activities of the individual compounds (SAIC) in both RL and GL, T-AOC (HPC-ABTS) was higher than the sum of the antioxidant activities of the individual compounds (SAIC) in both RL and GL. T-AOC (HPC-ABTS) was three-fold higher than SAIC in RL and two-fold higher than T-AOC (HPC-ABTS) in GL. T-AOC (HPLC-ABTS) was also nearly three-fold greater than SAIC in GL (Figure 4J). These results showed that the high antioxidant capacity of RL was associated with the high concentrations of flavonoids, and from the high antioxidant activities of individual flavonoids and flavonoid synergistic effects.

### Antioxidant activity in the blood of rats fed leaf extracts

**Figure 5:**
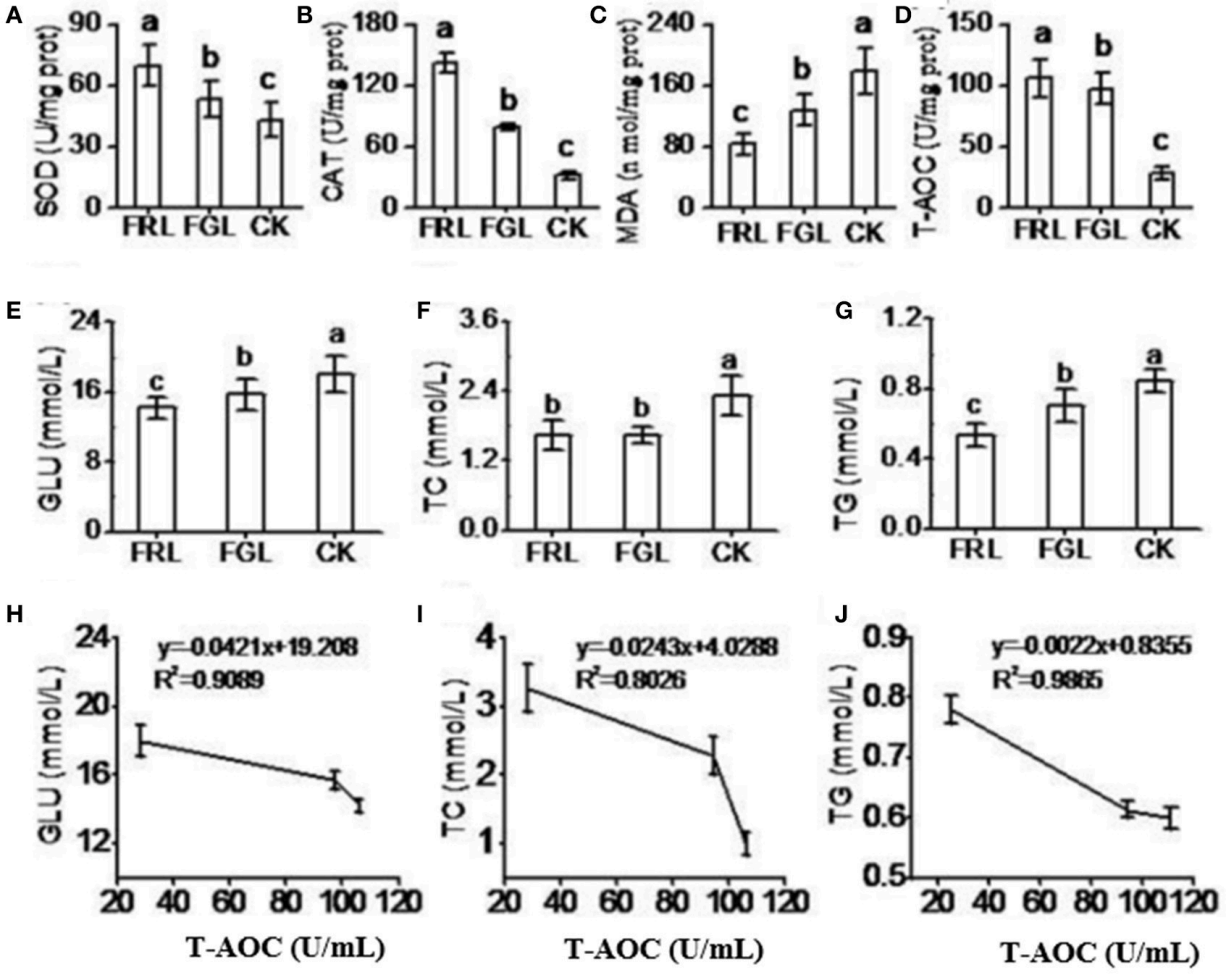
Antioxidant capacity in rat serum. **(A–G)** CK, the normal control group of rats receiving the basic diet and distilled water; FRL, the treatment group of rats receiving the basic diet and red leaf extract solution (50 mg/kg/day, dissolved in distilled water); FGL, the treatment group of rats receiving the basic diet and green leaf extract solution (50 mg/kg/day, dissolved in distilled water). GLU, blood glucose; TC, cholesterol; TG, triglyceride. **(H–J)** Regression analysis of T-AOC to GLU **(H)**, TC **(I)**, and TG **(J)** in rat serum. The values are expressed as the mean ± SD (*N* = 10/group). Lower-case letters indicate significance at *P* < 0.05 by Duncan's new multiple range test.

With the objective of introducing and producing a new nutrient, we conducted an animal model experiment to demonstrate that the crude extract of RL has powerful antioxidant capacity. The results indicated that the SOD and CAT activities and T-AOC remained significantly higher and the MDA content remained significantly lower in the serum of rat fed a diet with RL extract (FRL group) than in the serum of the rat fed a diet with GL extract (FGL group) or standard diet (CK group) (Figure 5). We also measured blood glucose (GLU), cholesterol (TC), and triglyceride (TG) levels in rat sera. Serum TC and TG levels were lower in the FRL group than in the FGL group (Figure 5). GLU, TC, and TG levels were negatively correlated with T-AOC (Figures 5E–G). These results indicated that consumption of RL extract might improve antioxidant capacity, decreasing pathological indices in rat blood.

## Discussion

Flavonoid compounds play important roles in various aspects of plant growth and development and have positive effects on human quality of life (Crozier et al., 2009). Their metabolism and function in plants are affected by not only environmental, but also inherent factors, such as transcriptional regulation and the interactions of the compounds with each other and their surroundings (Winkel-Shirley, 2001). Flavonoids are involved in defense strategies and signaling, particularly, in interactions between plants and their environment. Each class of flavonoid has been shown to have biological activity, epidemiological studies have revealed that flavonoids have antioxidant activities toward cancer, cardiovascular, and neurodegenerative diseases, or an anti-aging effect that is useful for application in cosmetic products (Grotewold, 2006; Boudet, 2007). As a chemical reaction system, the compositions and structures of flavonoid compounds vary greatly, and the interrelation of compounds is complex and diverse due to the metabolic flux and branches catalyzed by the enzyme system (Jorgensen et al., 2005). Further, the flavonoid compositions and contents of different plants vary depending on growth and developmental stages. This complex regulation includes top-down metabolic flux, bottom-up feedback regulation, and interactions among compounds, thus, different of flavonoids coordinate with each other in metabolism to form a flavonoids system. The flavonoid system is established through the interactions and interdependence of multiple compounds possessing special biological functions as well as the external plant environment. Understanding the biological function of the flavonoid system from a metabolic perspective and, importantly, explaining the effects of their intake are research goals that have been attracting increasing attention. Here, we attempted to establish a systematic, multistep approach to address these questions.

PCA implied that the synergistic effect of the flavonoid system played an important role in the antioxidant capacity of crabapple leaves. The percentage of PC_1_, the number of compounds distributed in the first quadrant, and the correlation among the first five compounds with high contribution rates to PC_1_ were all higher in RL than in GL. This result supported the conclusion that a significant cooperative relationship was involved in the flavonoid system in RL, whereas this relationship was inferior in GL. Furthermore, analysis of the diversity, evenness and dominance indices, combined with CA, revealed distinguishing characteristics of the flavonoid system at the leaf developmental stages of RL and GL. However, the leaves at certain stages, such as S6, stood out among the leaves from all other10 stages, displaying optimal flavonoid system characteristics in the two cultivars. This finding may provide guidance regarding the selection of optimal leaf materials for further study and application.

When tracing the origin of the synergistic antioxidant effect of the flavonoid system, the flavonoid biosynthesis pathway should be considered. A top-down flavonoid metabolic flux with positive or feedback regulation on the process exists (Yin et al., 2012), where flavonoid accumulation is controlled by structural genes (such as *CHS, F3H*, and *DFR*) and transcription factors (such as R2R3-MYBs) (Nakabayashi et al., 2014). *McMYB10*, an R2R3-MYB TF, is an activator of anthocyanin biosynthesis via regulating the genes *F3'H, DFR*, and *ANS* in red tissues (Tian et al., 2015), while *McMYB16* may be a repressor of red formation, as downregulation of the homologous *FcMYB1* in strawberry fruits induced the up-regulation of *ANS* and a strong repression of *ANR* and *LAR* transcript accumulation, resulting in increased anthocyanins and undetectable levels of flavan-3-ols (Salvatierra et al., 2013). *McMYB4*, homologous to *AtMYB111* of *Arabidopsis thaliana*, may participate in flavonol biosynthesis in crabapple. *AtMYB111* was reported to control flavonol accumulation through interaction with target genes, such as *FLS* and *UGT91A1* and *UGT84A1* (Pandey et al., 2014). In addition, slight *UFGT* expression was detected in GL, which differed from a report in white grapes, whose skin showed loss of *UFGT* expression (Boss et al., 1996) but was similar to the results of *UFGT* expression in white apple flesh and skin as well as red and green leaves (Espley et al., 2007). These results indicated that *UFGT* may be involved in the biosynthesis of other flavonoid glycosides, such as quercetin 3-O-β-D-glucuronide and flavone 3-O-glucoside (Xiao et al., 2014). Interestingly, luteolin-5-O-rutinoside was detected only in GL in this study. The reason might be the high FNS activity in GL and absence of FNS activity in RL, similar to the finding that red petals of *Gerbera Hybrida* show no FNS II expression while white petals do (Martens and Mithofer, 2005). The molecular mechanism by which *UFGT* regulates flavonoid glycoside biosynthesis and *FNS* regulates flavones (e.g., luteolin-5-O-rutinoside in GL) should be explored further.

The on-line HPLC-ABTS system provided fast, reliable and high-efficiency detection of the antioxidant capacity of individual compounds, as demonstrated by Shi et al. (2009). In the present study, we identified 12 negative peaks for 19 types of flavonoid compounds and demonstrated that T-AOC was higher than that indicated by the measurements of each compound (Supplementary Table S1), suggesting a synergistic effect among the compounds. However, using online HPLC-ABTS, we were unable to determine the antioxidant capacity of some compounds because no negative peaks appeared in P1, P4, P6, P7, P8, P10, and P18. The lack of negative peaks may have been due to the low content or low antioxidant capacity of these compounds. Alternatively, the corresponding negative peaks may not have been detected using HPLC-ABTS if the online reaction time was insufficient to allow reaction between ABTS+ and the antioxidants in the test system, such as the phloridzin (P18). Therefore, we transformed the phloridzin values determined by ultraviolet spectrophotometry to the values corresponding to the online analysis (Supplementary Table S1). The results indicated that this online system has potential for analyzing total and individual antioxidant capacities of functional compounds, but requires further improvement.

GLU, TC, and TG were lower, while SOD, CAT, and T-AOC activities were significantly higher, and MDA content was lower in the sera of treated rats than in those of non-treated control rats. This finding indicated that the enhancement of the antioxidant capacity helped reduce GLU, TC, and TG in rats fed RL extracts owing to some extent to the synergistic protective effect of total flavonoids (Lewis et al., 2013). Based on previous reports on the health benefits of flavonoids, these compounds should reduce fat accumulation and facilitate GLU movement as they participate in scavenging reactive oxygen species, inhibiting lipid peroxidation (which generates MDA), and promoting antioxidant defenses through the stimulation of antioxidative enzymes (Sugiura et al., 2012).

In view of the synergistic, it has been previously reported that, propolis induces a high bactericidal activity when consumed as a whole, while isolated compounds, shows low activity due to the lack of a stable and reproducible chemical composition. (Scheller et al., 1999; Bachiega et al., 2012). In addition, when foods are consumed together, the T-AOC may be modified via synergistic, additive, or antagonistic interactions among the constituents (Wang et al., 2011). Strong support for this conclusion was provided by our findings, which revealed that the T-AOC was significantly higher than the SAIC, with the synergistic effects being stronger in RL than in GL. Meanwhile, the antioxidant capacities of most of the individual compounds in RL were superior to those in GL. These findings could be explained by the synergy being a result of both interactive effects among and individual effects of the flavonoids compounds.

The systemic approach we established for comparing differences in the flavonoid system between cultivars, which describes the combined principles for deep analysis of results, is a novel approach compared to systems theory (Bertalanffy, 1968). Based on our findings, we recommend the application of this approach for other compounds in plant secondary metabolism and even in the areas of nutrient quality composition analysis in food and quantitative trait analysis in plant breeding.

## Materials and methods

### Plant materials

Leaves were collected from 4-year-old *Malus* cv. “Royalty” and “Flame” trees; grafted on *Malus* hupehensis and planted in the Crabapple Germplasm Resource Garden of BUA, Beijing, China. On June 10, 2013, when the annual shoots stopped growing, we randomly selected 10–20 annual shoots with similar growth vigor from the south side of each plant and collected 10 leaves from the tip to the base nodes of each shoot. The leaves representing the 10 developmental stages, were labeled S1 to S10. The weight at S1 was 0.1–0.2 g and that at S10 was 0.8–1.0 g, and the width of the collected leaves at the same stage was similar for each cultivar. The experiment was performed using a randomized block design of two cultivars with three biological replicates, and five trees were used in each plot. After collection, 30–40 leaf discs (1 cm diameter, punched in the middle of each side of the main vein) were collected randomly from leaves at each stage; per plot, frozen in liquid nitrogen, and stored at −80°C for compound analysis and RNA extraction. The remaining non-perforated leaves were stored at −20°C for enzyme analysis and animal experiments.

### Animals and experimental design

Weaning male Sprague-Dawley rats (7-week-old, 180–200 g) were purchased from Beijing Xinglong Experimental Animal Farms and kept five per cage under standard housing conditions (12/12 h light/dark cycle, lights on at 7:00 a.m., humidity 50–60%, and at 22 ± 2°C), with *ad libitum* access to food and water throughout the study period. One week after acclimatization to a basic diet (containing crude protein ≥ 20%, crude ash ≤ 8%, crude fiber ≤ 5%, 1.8% ≥ calcium ≥ 1%, 1.2% ≥ phosphorus ≥ 0.6%, lysine acuity ≥ 1.35%, salt = 1.35%; purchased from Beijing Keao Xieli Feed Company Limited), the rats were randomly divided into three groups of six rats per group: Group 1 (CK), the normal control group received basic diet and distilled water; Group 2 (FRL), received basic diet and RL extract (50 mg/kg/day, dissolved in distilled water); and Group 3 (FGL), received basic diet and GL extract (50 mg/kg/day, dissolved in distilled water).

At the end of the 5-week experimental period, after 12 h of starvation, all rats were anesthetized with intraperitoneal chloral hydrate (300–350 mg/kg) at the same time of the day to avoid circadian metabolic fluctuations. Blood samples were obtained from the carotid artery and centrifuged immediately at 800 × g for 15 min at −4°C to separate the serum samples (Jia et al., 2014). All study procedures performed on the animals were approved by the Animal Care and Use Ethics Committees of China Agricultural University, and all rats received humane care in accordance with the guidelines.

### Preparation of leaf extracts for the rat diet and extraction of flavonoids

To prepare the diet, RL and GL at S6 were extracted. Samples were extracted using an 80% methanol solution and a material-to-solvent ratio of 1:10 under sonication at a frequency of 12,000 Hz at 45°C. Extraction was performed three times in total for 2.0, 1.5, and 1.0 h. Next, the solutions were filtered and concentrated at 40°C with a rotary evaporator under reduced pressure before being frozen at low temperature in a ModulyoD-230 freeze dryer (Thermo Fisher, New York, USA) to obtain a powder. Approximately 50 g of leaf extract powder per sample type (RL and GL) was added to the rats' diet.

For flavonoid extraction, 1.0 g of frozen RL and GL was powdered in liquid nitrogen using a mortar and pestle. Then, 3 ml of methanol/water/formic acid (80:19:1, v/v/v) was added to the sample, followed by 50 min of sonication at 12,000 Hz at 45°C to extract the flavonoid compounds. The compounds were filtered through a 0.22-μm membrane (Shanghai ANPEL, Shanghai, China) before HPLC-DAD and HPLC-MS analyses. We used mixed leaf samples from S1 to S10 for the identification of flavonoid types and for the detection of temporal changes in the leaf flavonoid composition during leaf development. We used leaf samples at S6 for further experimental analyses after identifying it as the optimal stage.

### HPLC-ESI (±)-MS^2^ analysis and identification of flavonoids

For HPLC-ESI-MS analyses of the flavonoids, we followed the method of Yi et al. (2016) with modification. Experimental standards of cyanidin-3-O-galactoside, quercetin-3-O-glucoside, astilbin, phloridzin, rutin, quercetin-3-O-arabinoside, quercetin, and luteolin were purchased from Sigma-Aldrich (Steinheim, Germany); experimental standards of 4-O-coumaroyl quinic acid, procyanidin B2, (-)-epicatechin, and taxifolin-3-O-glucoside were purchased from Sigma-Aldrich (Poole, U.K.); data obtained for experimental standards and information regarding the identified compounds were combined to identify the flavonoids.

### HPLC-DAD analysis

An Agilent 1100 series HPLC system (Agilent Technologies, Wilmington, DE, USA) was used in our experiments. For chromatographic separation, eluent A was 0.5% aqueous formic acid, eluent B was 100% acetonitrile, and the elution gradient was as follows 90% A at 0 min, 90% A to 81% A from 0 to 40 min, 81% A to 60% A from 40 to 50 min, 90% A at 50.01 min, and 90% A at 60 min. The flow rate was 1.0 ml/min, and injection volume was 20 μl. The ZORBAX Eclipse XDB-C18 (5 μm, 150 × 4.6 mm, Agilent, USA) analytical column temperature was 27°C for all analyses. Chromatograms were acquired at 520 nm for anthocyanins and at 350 and 280 nm for all other flavonoids. DAD data were recorded from 200 to 600 nm.

### Quantitative analysis of individual flavonoid compounds

We used quantitative analysis of multi-components by single-marker (QAMS) to calculate the contents of phytochemical products in the leaves (Zhou et al., 2008; Hou et al., 2011). We analyzed eight classes of flavonoid compounds as follows. For semi-quantification of the anthocyanins, a standard curve was drawn from 0 to 200 μg/g with a standard compound of cyanidin-3-O-galactoside at 20 μg/g. The equation of the calibration curve describing the results was Y_1_ (equivalent content μg/g) = (0.003^*^A_1_+0.3)/m (m ≤ 1.0 g, *R*^2^ = 0.9999), where A_1_ is the peak area at 520 nm. For the semi-quantification of flavonol and phenolic acid, a standard curve was drawn from 0 to 3,000 μg/g with a standard compound of quercetin at 300 μg/g. The equation for the calibration curve describing the results was Y_2_ (equivalent content μg/g) = (0.02^*^A_2_-10.6)/m (m ≤ 1.0 g, *R*^2^ = 0.9996), where A_2_ is the peak area at 350 nm. For semi-quantification of the dihydrochalcone, proanthocyanidin, dihydroflavonol, flavones, and flavan-3-ols, a standard curve was drawn from 0 to 2,000 μg/g with a standard compound of phloridzin at 200 μg/g. The equation of the calibration curve describing the results was Y_3_ (equivalent content μg/g) = (0.0005^*^A_3_-0.05)/m (m ≤ 1.0 g, *R*^2^ = 0.9998), where A_3_ is the peak area at 280 nm.

### Antioxidant activity and other physical characteristics of leaf samples at S6 *in vivo* and *in vitro*

#### On-line HPLC-ABTS assay

HPLC coupled with the ABTS assay was performed using the method (Arnao et al., 2001), with some modifications. ABTS [2, 2′-azino-di-(3-ethyl-benzothiazoline-6-sulphonic acid)] was purchased from Sigma-Aldrich (St Louis, MO, USA). A stock solution containing 140 mM potassium persulphate and 7 mM ABTS was prepared and kept at room temperature in darkness for 12–16 h to stabilize the radicals. The radical reagent was prepared by diluting the stock solution with absolute ethyl alcohol to an absorbance of 0.70 ± 0.02 at 734 or 414 nm. The S6 leaf sample was extracted in the same way as in the HPLC analysis [1.0 g frozen leaves (RL and GL) were extracted with 3 ml of methanol/water/formic acid (80:19:1, v/v/v)]. The HPLC-ABTS system used consists of a Waters e2695 HPLC separation system, post-column reaction system, Waters e2998 photodiode array detector (PAD), and Waters 2489 UV/Visible detector. We used the same ZORBAX Eclipse XDB-C18 (5 μm, 150 × 4.6 mm, Agilent, USA) analytical column in an Agilent 1100 HPLC system to separate the compounds from the samples. The HPLC separation conditions were the same as above to maintain the same retention time. The HPLC eluates from the column arrived at a T-junction, where the ABTS reagent was added, at 0.4 ml/min by a Waters Reagent Pump (Waters Corporation, Massachusetts, USA). After the eluates were mixed with ABTS reagent in a reaction coil (15 m, 0.25 mm i.d. PEEK tubing), a Waters temperature control module (Waters Corporation, Massachusetts, USA) maintained the temperature at 40°C. The negative peaks were measured by DAD at 517 nm. We replaced the Milli-Q water with ABTS^+^ as the control. The data were analyzed using the Waters ChemStation Software.

#### Analysis of the T-AOC values of mixed compound samples by an on-line HPLC-ABTS system

In the on-line HPLC-ABT system, we used a Waters X-Terra MS C18 guard column (to hold the samples without separation of the compounds) instead of the ZORBAX Eclipse XDB-C18 (5 μm, 150 × 4.6 mm, Agilent, USA) analytical column to analyze the T-AOC (HPLC-ABTS) of the mixed compound samples. For a given sample, we diluted the extract solution five-fold for RL and three-fold for GL because the antioxidant activities of the undiluted extract solution from 1.0 g of leaf samples appeared as a flat negative peak using the UV/Visible chromatographic detector.

#### Detection of antioxidant capacity relative to that of ascorbic acid (Vc) of phloridzin and other compounds

Phloridzin is prevalent in *Malus* plants and has been reported to have antioxidant activity both *in vitro* and *in vivo* (Jugde et al., 2008; Li et al., 2014). However, the on-line HPLC-ABTS system has certain shortcomings or limitations. On one hand, the on-line post-column reaction time was set shortly (most < 1 min) that might cause false-negative results for antioxidants with slow kinetics of reactions (Lu et al., 2017). For example, phloridzin as an excellent antioxidant, may stay in the reaction coil for longer than the limited time in the system, preventing its detection, its negative peak could not be detected in on-line ABTS analysis. On the other hand, the post-column reaction system consisted of a post-column reaction model (peak reaction coil) in which the antioxidants reacted with the free radicals, and the reaction might be not complete in a small coil. Therefore, we used a Rapid ABTS method (Total Antioxidant Capacity Assay Kit, Nanjing Jianchen Bioengineering Institute, Nanjing, China) to detect the antioxidant capacities of phloridzin and ascorbic acid (Vc) by A414 ELISA in an off-line system. After a 10 mM Trolox standard solution was diluted to 0.15, 0.3, 0.6, 0.9, 1.2, and 1.5 mM, and a linear equation for the absorbance/concentration ratio was established, the absorbance values of 1.0 mM phloridzin and of Vc were each determined relative to the Trolox-equivalent antioxidant capacity. We detected the concentrations of the 1.0 mM Vc sample negative peak area using the HPLC-ABTS system. According to the method described above, the values for other compounds relative to the Vc antioxidant values were calculated by the formula: relative to Vc = (each compound negative peak area/each compound positive peak area)/(Vc negative peak area/ Vc positive peak area).

#### Determination of SOD and, CAT activities, MDA, and T-AOC

All enzyme extractions were conducted at 4°C. Each sample (0.1 g fresh weight, at S6) was thoroughly homogenized in 1.0 ml of 50 mM phosphate buffer (pH 7.8) containing 0.1 mM ethylene diaminetetraacetic acid (EDTA) and 0.05 g of quartz for homogenization. The homogenates for SOD, CAT and MDA were centrifuged at 8,000 × g for 10 min; and the homogenate for T-AOC was centrifuged at 10,000 × g for 10 min. The crude supernatant was used to determine the SOD and CAT activities, total antioxidant capacity (T-AOC) was measured using the ferric reducing activity of plasma (FRAP) assay, and MDA concentrations in each tissue, using commercial kits (Product Codes: FY1, FY2, EY2-1, FG5, respectively; Suzhou Comin Biotechnology Co., Ltd, Jiangsu, China). For measuring these four parameters in animal serum samples (centrifuged immediately at 800 × g for 15 min at 4°C from blood samples), the above kits were also used.

#### Determination of glucose (GLU), TC, and TG levels *in vivo*

The serum level of GLU was measured according to a standard procedure using a Vet Test 8008 Automatic Biochemical Analyzer (IDEXX, USA). TC and TG were measured using a Hitachi 7020 Automatic Analyzer (Hitachi, Tokyo, Japan).

### RNA extraction and real-time PCR analysis

Total RNA was extracted from leaves at S6 and used and real-time PCR analysis following by Yi et al. (2016). The primers used are listed in Supplementary Table S2. PCRs were carried out for 40 cycles of 95°C for 15 s and 59°C for 20 s and were run with three biological replicates and two technical replicates. Gene expression levels were calculated using the 2^(−ΔΔCt)^ method. *Malus 18S ribosomal* RNA gene (GenBank accession number: DQ341382) was used as a reference gene because of its consistent transcript level among “Royalty” and “Flame” leaves.

### Statistical analysis

For diversity analysis of flavonoid systems in both leaf types, we created matrices of the values of the measured content of each type of flavonoid compound at different developmental stages for RL and GL as well as the calculated Shannon-Wiener diversity index, the evenness index and Simpson's index according to the methods described by Magurran (2003) and Morris et al. (2014). The Shannon-Wiener diversity index (H) was applied to measure the diversity of the flavonoid systems: H' = $-\sum_{\text{i}}^{\text{s}}{\text{P}}_{\text{i}}\ln\;{\text{P}}_{\text{i}},\text{i}=1,2,3,\ldots \text{s},{\text{p}}_{\text{i}}={\text{n}}_{\text{i}}/\text{N},$ where n_i_ is the total amount of the ith type of flavonoid compound, N is the total amount of all types of flavonoid compounds, and s is the number of types of flavonoid compounds in the sample. The Simpson index (D) was used to calculate the dominance index for each type of flavonoid compound: $D=-\sum_{\text{i=1}}^{\text{s}}\frac{{\text{n}}_{\text{i}}\;\left({\text{n}}_{\text{i}}-\text{1}\right)}{\text{N }\left(\text{N}-\text{1}\right)}$ where n_i_ is the amount of the *i*th type of flavonoid compound, and N is the total amount of all types of flavonoid compounds. The evenness (E) of the types of flavonoid systems was measured using the formula E = H'/ln S, where H' is the Shannon-Wiener diversity index, and S is the amount of all types of flavonoid compounds.

Clustering analysis (CA): CA is the most similar objects are grouped first, as the similarity decreases, all subgroups are fused into a single cluster (Simao et al., 2010). We employed system CA using the diversity index (H), dominance (D), and evenness index (E) as the indices using the SPSS 17.0 software (SPSS Inc., Chicago, IL, USA). We obtained clustering results for diversity in the flavonoid system of the leaves at 10 developmental stages using the Euclidean shortest distance method.

Principal component analysis (PCA): PCA convert a set of correlated variables into a set of values of linearly uncorrelated variables (Patras et al., 2011). The distributions of the compounds in RL and GL were then drawn in PCA diagrams using SPSS 17.0. These diagrams revealed the percentage variations of PC_1_ and PC_2_ in flavonoid systems, the contribution rates of individual compounds to PC_1_ and PC_2_, and the interrelations of the individual flavonoid compounds.

The data are presented as the mean ± standard deviation (*SD*). Analysis of variance (ANOVA, SPSS 17.0 software, SPSS Inc., Chicago, IL, USA) of all values was used to assess differences in the means among samples (*P* < 0.05). Duncan's multiple analysis and Student's *t*-test were used to identify significant differences among groups (*P* < 0.05, *P* < 0.01). Graphs were prepared in Origin Pro 8.0 SR4 (Origin Lab, Northampton, MA, USA) and Microsoft Office PowerPoint 2007.

## Author contributions

YY: designed the study; XQ and YL: performed the research; SF: provided new methods and materials; XQ, YL, and ZP: wrote the manuscript; and all authors approved the revisions.

### Conflict of interest statement

The authors declare that the research was conducted in the absence of any commercial or financial relationships that could be construed as a potential conflict of interest.
