# Supplementary material for: Systematic Chemical Analysis Approach Reveals Superior Antioxidant Capacity via the Synergistic Effect of Flavonoid Compounds in Red Vegetative Tissues
Source: Front Chem. 2018 Feb 2;6:9. doi: 10.3389/fchem.2018.00009 (PMC5808280; doi:10.3389/fchem.2018.00009)
Supplement: Supplementary file 1 [file DataSheet1.docx]

**Systematic Chemical Analysis Approach Reveals Superior Antioxidant Capacity via the Synergistic Effect of Flavonoid Compounds in Red Vegetative Tissues**

Xiaoxiao Qin^,†^, Yanfen Lu^, †^, Zhen Peng, Yuncong Yao^,*^, Shuangxi Fan^,*^

*Beijing Key Laboratory of New Technology in Agricultural Application, National Demonstration Center for Experimental Plant Production Education, Beijing Collaborative Innovation Center for Eco-Environmental Improvement with Forestry Fruit Trees, Beijing University of Agriculture, Beijing, China*

^†^ Contributed equally to this work.

^*^ **Corresponding author:**

Yuncong Yao, Shuangxi Fan, Department of Plant Science and Technology, Beijing University of Agriculture, Beijing, 102206, China; Email: yaoyc_20@126.com, [fsx20@163.com](mailto:fsx20@163.com), Tel: +86-10- 807 99000, Fax: +86-10-80799004;

**Type:** original research

**Supplementary Text S1**

The identification of the compounds:

In the study, we employed HPLC-ESI(±)-MS^2^ analysis to identify the kinds of compounds by standards and comparing their spectroscopic data to literature, the known 19 compounds listed in Table 1.

Compounds1: Quercetin-3-O-diglucoside (P1)

Based on [M-H] and MS^2^[M-H]^-^(m/z) signals, the compound quercetin-3-O-diglu coside was identified by produced precursor ion with the [M-H]^-^ at m/z 625, and with fragments at m/z 463 and 301,which were evidently produced by the loss of the one glucoside and two glucoside respectly. This compounds was described by Sánchez et.al ([SánchezRabaneda, Jauregui, Lamuela‐Raventós, Viladomat, Bastida & Codina, 2004](#_ENREF_6)).


Compounds2: Cyanidin-3-O-galactoside (P2)

Cyanidin-3-O-galactoside was identified by a peak at R_t_ 5.6 min based on the retention time and molar mass with the [M-H]+ at m/z 449 and fragments with the [M-H-162]^+^at m/z 287 of the standard substance, this compounds was described by Sánchez et.al ([SánchezRabaneda *et al.*, 2004](#_ENREF_6)).


Compounds3: 4-O-Coumaroyl quinic acid (P3)

4-O-Coumaroyl quinic acid was detected and identified based on stands data indicating that the compound had a mass spectral signal [M-H]^-^ at m/z 353 and fragmentation of the negatively charged molecular ion at m/z 191[M-H-162]^-^,173 [M-H-162-18]^-^ in the HPLC-MS analysis and it had been reported in apples fruits ([Awad, de Jager & van Westing,2000](#_ENREF_2)).


Compounds 4: Taxifolin-3-O-glucoside (P4)

Taxifolin-3-O-glucoside was identified by the stands and its had a [M–H]^-^ at m/z 465 and an MS^2^ ion at m/z 285[M-H-162-18]^-^，241[M-H-162-18-44]^-^ and the taxifolin had been reported in apples ([Vega-Villa, Remsberg, Ohgami, Yáñez, Takemoto, Andrews & Davies, 2009](#_ENREF_7)).

Compounds 5: Procyanidin B2

Procyanidin B2 was identified by the stands and identified with precursor ion [M-H]^-^ at m/z 577, fragmentation with m/z 451[M-H-126]^-^，425[M-H-152]^-^，407[M-H-170]^-^.

Compounds 6: Xyloside roseoside (P6)

Xyloside roseoside was identified by its precursor ion [M–H]^-^ at m/z 517 and the fragmentation MS^2^ ion at m/z 385[M-H-132]^-^ and it indicated the precursor ion lost one xylose, the compound roseoside was ever reported in *Malus*  leaves ([Winterhalter, Güldner, Jakob & Schreier, 1994](#_ENREF_8)).

Compounds 7: Astilbin (P7)

Astilbin was identified by the stands and the precursor ion [M–H]^-^ at m/z 449 and the fragmentation MS^2^ ion at m/z 269[M-H-162-18]^-^.

Compounds 8: Roseoside (P8)

Roseoside was identified by the precursor ion [M–H]^-^ at m/z 385 and the fragmentation MS^2^ ion at m/z 223[M-H-162]^-^ and it was ever reported in *Malus* leaves ([Winterhalter *et al.*, 1994](#_ENREF_8)).

Compounds 9: (-)-Epicatechin (P9)

(-)-Epicatechin was identified by the stands and the fragmentation of the negatively charged precursor ion [M-H]^-^ at m/z 289, fragmentation with m/z 245[M-H-44]^-^, 205[M-H-84]^-^ and it had been reported in apples juice ([Berregi, Santos, del Campo & Miranda, 2003](#_ENREF_3)).

Compounds 10: Rutin (P10)

Rutin was identified by the stands and the fragmentation of the negatively charged precursor ion [M-H]^-^ at m/z 609, fragmentation with m/z 565[M-H-44]^-^，301[M-H-162-146]^-^, This compounds was described by Sánchez et.al ([Sánchez, Rabaneda *et al.*, 2004](#_ENREF_6)).

Compounds 11: Quercetin-3-O-glucoside (P11)

Quercetin-3-O-glucoside was identified by the stands and the fragmentation of the negatively charged precursor ion [M-H]^-^ at m/z 463, fragmentation with m/z 301[M-H-162]^-^.

Compounds 12: Quercetin-7-O-glucoside (P12)

Quercetin-7-O-glucoside was identified by the fragmentation of the negatively charged precursor ion [M-H]^-^ at m/z 463, fragmentation with m/z 301[M-H-162]^-^ and it judeged by the structural characterization of flavonol 3,7-di-O-glycosides reported by Ablajan ([Ablajan, Abliz, Shang, He, Zhang & Shi, 2006](#_ENREF_1)).

Compounds 13: Quercetin-3-O-arabinoside (P13)

Quercetin-3-O-arabinoside was identified by the stands and the fragmentation of the negatively charged precursor ion [M-H]^-^ at m/z 433, fragmentation with m/z 301[M-H-132]^-^.

Compounds 14: Acetyl quercetin-3-O-glucoside (P14)

Acetyl quercetin-3-O-glucoside was identified by the fragmentation of the negatively charged precursor ion [M-H]^-^ at m/z 505, fragmentation with m/z 463[M-H-132]^-^ and m/z 301[M-H-132]^-^, it indicated that the precursor ion lost one acetyl group and lost one acetyl group, a glucoside in the meantime.

Compounds 15: Acetyl quercetin-7-O-glucoside (P15)

Acetyl quercetin-7-O-glucoside was identified by the fragmentation of the negatively charged precursor ion [M-H]^-^ at m/z 505, fragmentation with *m/z* 463[M-H-132]^-^ and m/z 301[M-H-132]^-^ and it judeged by the structural characterization of flavonol 3,7-di-O-glycosides reported by Ablajan ([Ablajan *et al.*, 2006](#_ENREF_1)).

Compounds 16: Quercetin-7-O-arabinoside (P16)

Quercetin-7-O-arabinoside was identified by the fragmentation of the negatively charged precursor ion [M-H]^-^ at m/z 433, fragmentation with m/z 301[M-H-132]^-^ and it judeged by the structural characterization of flavonol 3,7-di-O-glycosides reported by Ablajan ([Ablajan *et al.*, 2006](#_ENREF_1)).

Compounds 17: Quercetin-3-O-rhamnoside (P17)

Quercetin-3-O-rhamnoside was identified by the fragmentation of the negatively charged precursor ion [M-H]^-^ at m/z 447, fragmentation with m/z 301[M-H-146]^-^ and it had been reported in apples ([Lommen, Godejohann, Venema, Hollman & Spraul, 2000](#_ENREF_5)).

Compounds 18: Phloridzin (P18)

Phloridzin was identified by the stands and the fragmentation of the negatively charged precursor ion [M-H]^-^ at m/z 435, fragmentation with m/z 273[M-H-162]^-^, This compounds was described by Sánchez et.al ([SánchezRabaneda *et al.*, 2004](#_ENREF_6)).

Compounds 19: Luteoline-5-O-rutinoside (P19)

Luteoline-5-O-rutinoside was calculated by the stands luteoline mass spectrometry signal and this compounds had ever been reported in apples ([SánchezRabaneda *et al.*, 2004](#_ENREF_6)). Finally, luteoline-5-O-rutinoside was identified by the fragmentation of the negatively charged precursor ion [M-H]^-^ at m/z 593, fragmentation with m/z 285[M-H-162-146]^-^, luteolin had been reported in *Malus* plants ([Kislichenko & Novosel, 2007](#_ENREF_4)) .

**References:**

Ablajan K., Abliz Z., Shang X.-Y., He J.-M., Zhang R.-P. & Shi J.-G. (2006) Structural characterization of flavonol 3, 7-di-O-glycosides and determination of the glycosylation position by using negative ion electrospray ionization tandem mass spectrometry. *Journal of Mass Spectrometry*, **41**, 352-360.

Awad M.A., de Jager A. & van Westing L.M. (2000) Flavonoid and chlorogenic acid levels in apple fruit: characterisation of variation. *Scientia Horticulturae*, **83**, 249-263.

Berregi I., Santos J.I., del Campo G. & Miranda J.I. (2003) Quantitative determination of (-)-epicatechin in cider apple juices by 1 H NMR. *Talanta*, **61**, 139-145.

Kislichenko V. & Novosel E. (2007) Flavonoids from leaves of Pyrus communis, Malus sylvestris, and Malus domestica. *Chemistry of Natural Compounds*, **43**, 704-705.

Lommen A., Godejohann M., Venema D., Hollman P. & Spraul M. (2000) Application of directly coupled HPLC-NMR-MS to the identification and confirmation of quercetin glycosides and phloretin glycosides in apple peel. *Analytical chemistry*, **72**, 1793-1797.

SánchezRabaneda F., Jauregui O., Lamuela‐Raventós R.M., Viladomat F., Bastida J. & Codina C. (2004) Qualitative analysis of phenolic compounds in apple pomace using liquid chromatography coupled to mass spectrometry in tandem mode. *Rapid Communications in Mass Spectrometry*, **18**, 553-563.

Vega-Villa K.R., Remsberg C.M., Ohgami Y., Yáñez J.A., Takemoto J.K., Andrews P.K. & Davies N.M. (2009) Stereospecific high-performance liquid chromatography of taxifolin, applications in pharmacokinetics, and determination in tu fu ling (*Rhizoma smilacis glabrae*) and apple (*Malus*× *domestica*). *Biomedical Chromatography*, **23**, 638-646.

Winterhalter P., Güldner A., Jakob U. & Schreier P. (1994) 3-hydroxy-5,6-epoxy-β-ionyl-β-D-glucopyranoside from Malus domestica Leaves. *Natural Product Letters*, **4**, 57-60.

**Supplementary figures**

**
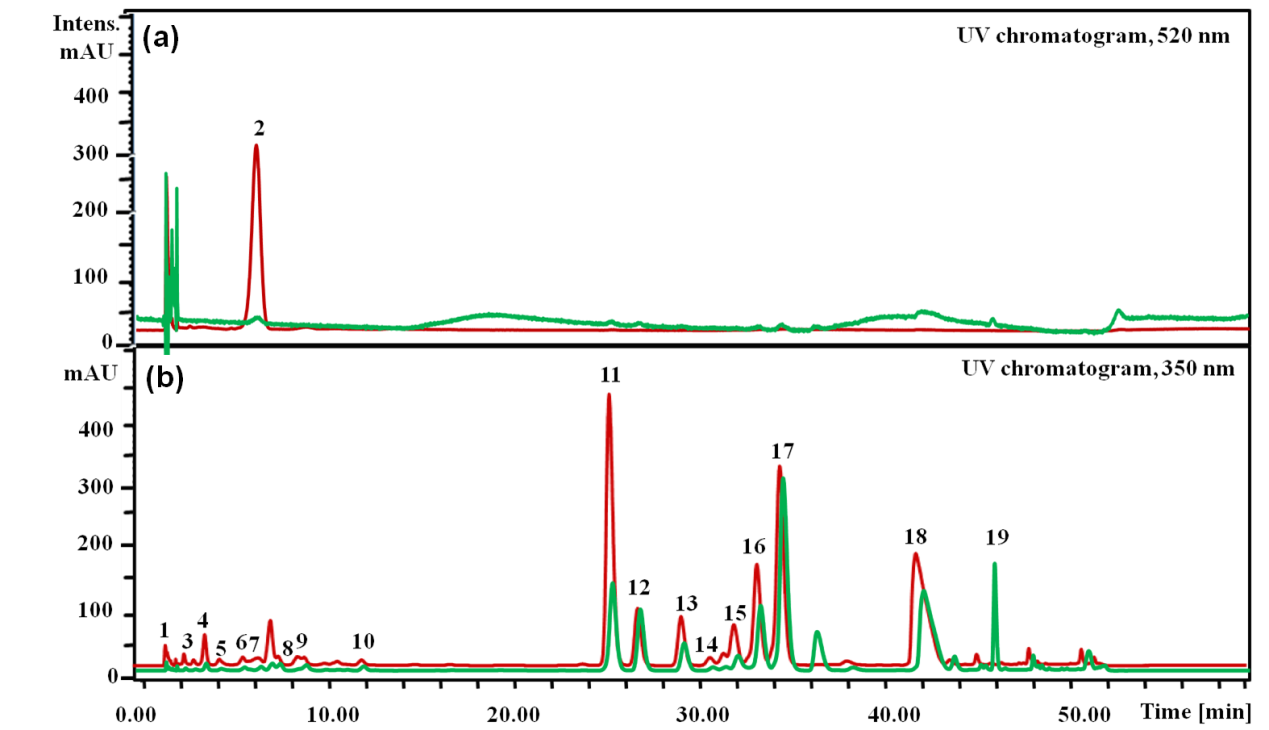
**

Supplementary Fig. S1 Composition of compounds in crabapple leaves as determined by HPLC-DAD analysis. (a) and (b) are chromatograms acquired at 520 nm and 350 nm, respectively. The red and green lines represent peaks from the ever-red (RL) and evergreen leaves (GL).

Supplementary Fig. S2 Contents dynamic changes of 19 compounds in ever-red (RL) and ever-green (GL) leaves during the developmental stages.

Supplementary Table S1 Antioxidant capacity of the main phenolic compounds in crabapple leaves.

| **Compounds Antioxidant capacity Antioxidant Coefficient Relative to Vc**  **RL GL RL GL RL GL** | | | | | | |
| --- | --- | --- | --- | --- | --- | --- |
| P1 | ND | ND | ND | ND | ND | ND |
| P2 | 67.89±8.32^c**^ | ND | 0.45±0.053 | ND | 1.98±0.46 | ND |
| P3 | 20.40±6.13^e**^ | 14.42±2.87^cdef^ | 0.15±0.028 | 0.1±0.015 | 8.94±0.4 | 7.21±0.32 |
| P4 | 40.40±6.13^d**^ | ND | ND | ND | ND | ND |
| P5 | 17.06±3.19^ef**^ | 3.16±0.41^f^ | 0.02±0.0048 | 0.003±0.00023 | 4.18±0.37 | 3.43±0.41 |
| P6 | ND | ND | ND | ND | ND | ND |
| P7 | ND | ND | ND | ND | ND | ND |
| P8 | ND | ND | ND | ND | ND | ND |
| P9 | 18.46±3.55^e*^ | 13.46±3.55 | 0.2±0.017 | 0.03±0.0024 | 3.81±0.21 | 3.24±0.19 |
| P10 | ND | ND | ND | ND | ND | ND |
| P11 | 48.03±3.91^d**^ | 27.83±5.75^c^ | 6.15±0.89 | 2.15±0.24 | 1.81±0.2 | 1.76±0.05 |
| P12 | 17.83±2.31^ef^ | 22.99±3.23^cd*^ | 1.89±0.13 | 2.45±0.36 | 1.89±0.29 | 2.45±0.14 |
| P13 | 13.53±2.89^ef^ | 10.10±2.44^def^ | 1.5±0.17 | 0.79±0.13 | 2.5±0.76 | 2.29±0.63 |
| P14 | ND | ND | ND | ND | ND | ND |
| P15 | 6.64±0.84^f^ | 8.79±0.96^ef*^ | 0.76±0.023 | 0.23±0.068 | 1.76±0.31 | 1.23±0.12 |
| P16 | 144.28±21.87^b**^ | 92.94±13.99^b^ | 18.74±3.73 | 12.79±2.34 | 10.74±1.49 | 9.79±1.52 |
| P17 | 16.73±3.76^ef^ | 17.29±2.18^cde^ | 2.25±0.92 | 2.34±0.89 | 0.49±0.13 | 0.54±0.22 |
| P18 | ND | ND | 10.03±2.32 | 7.29±1.46 | 5.91±0.98 | 5.67±0.69 |
| P19 | ND | 1.05±0.07^f**^ | ND | 0.04±0.01 | 0 | 0.04±0.006 |
| T-AOC | 245.86±20.03^a**^ | 190.45±18.24^a^ | - | - | - | - |
| VC | ND | ND | ND | ND | 1 | 1 |

(NPA*10^5^) stands negative peak areas. Antioxidant Coefficient (μmol) = flavonoids content (μg/g) **/** M * Relative to Vc; Relative to Vc = (each compound negative peak areas/each compound positive peak areas)/ (Vc negative peak areas/Vc positive peak areas). Lower-case letter indicated a significance at P＜0.05 by Duncan’s new multiple range test. ****** and ***** indicated significance at P＜0.01 and P＜0.05 by t test respectively.

Supplementary Table S2 Primer sequences used in this study.

| **Accession number** | **ID** | **Sequence (5’-3’)** | **Product length** | **Purpose** |
| --- | --- | --- | --- | --- |
| FJ599763 | McCHS-F | TGACCGTCGAAGTTCGC | 182 bp | qRT-PCR |
|  | McCHS-R | TTTGTCACACATGCGCTGGA |  |  |
| FJ817485 | McCHI-F | AGGAGTTGTCGGAGTCCGTT | 115 bp | qRT-PCR |
|  | McCHI-R | ACTTTCTCAGAGTATTGCTGGCC |  |  |
| FJ817486 | McF3H-F | ACGAAGACGAGCGTCCAAAG | 233 bp | qRT-PCR |
|  | McF3H-R | CTCCTCCGATGGCAAAGCAA |  |  |
| KF481684 | McF3’H-F | CGTTGCTGTCGCTCACGGATGA | 108 bp | qRT-PCR |
|  | McF3’H-R | ATGACGTGTCAGTGCCAGCTGTG |  |  |
| FJ817487 | McDFR-F | CCGAGTCCGAATCCGTTTGT | 126 bp | qRT-PCR |
|  | McDFR-R | CCTTCTTCTGATTCGTGGGGT |  |  |
| FJ817488 | McANS-F | CACAGGGGCATGGTGAACAA | 202 bp | qRT-PCR |
|  | McANS-R | TTCACTTGGGGAGCAAAGCC |  |  |
| KF711858 | McUFGT-F | TGGGCGGACACCAATCA | 194 bp | qRT-PCR |
|  | McUFGT-R | ATGTCTCCACCGCACCA |  |  |
| KF495602 | McFLS-F | ACGAGCAACCGGGAATCACAACTG | 120 bp | qRT-PCR |
|  | McFLS-R | CCCAGTTGGAGCTGGCCTCAGTA |  |  |
| DQ341382  JX013493 | 18S RNA-F | GTCACTACCTCCCCGTGTCA | 102 bp  161bp | qRT-PCR  qRT-PCR |
|  | 18S RNA-R  McMYB4-F  McMYB4-R | GAGCCTGAGAAACGGCTACC  GGACCAGCAGCAGGAAACTA  ACAACCCTCCATTAATGCCGAC |  |  |
| JX162681  KJ126856 | McMYB10-F | ACGCCACCACAAACGTCGTCG | 220 bp  139bp | qRT-PCR  qRT-PCR |
|  | McMYB10-R  McMYB16-F  McMYB16-R | GGCGCATGATCTTGGCGACAGT  GCTCACACCAACAAAGGAGC  GCAGCTCTTCCCACATCGAA |  |  |
